# ‘*Life became harder with COVID-19*’: exploring the experiences of the COVID-19 pandemic among youth living in eThekwini district, South Africa

**DOI:** 10.1186/s12889-024-19238-7

**Published:** 2024-07-17

**Authors:** Kalysha Closson, Erica Dong, Bongiwe Zulu, Janan J. Dietrich, Campion Zharima, Julie Jesson, Tatiana Pakhomova, Mags Beksinska, Angela Kaida

**Affiliations:** 1 Faculty of Health Sciences, Simon Fraser University, Burnaby, BC Canada; 2 Center of Gender Equity and Health, University of California San Diego, La Jolla, CA United States; 3 Wits MRU (MatCH Research Unit), Department of Obstetrics and Gynaecology, Faculty of Health Sciences, University of the Witwatersrand, Durban, South Africa; 4 Center for Epidemiology and Research in POPulation Health (CERPOP), Inserm, Université de Toulouse, Université Paul Sabatier, Toulouse, France; 5 Perinatal HIV Research Unit (PHRU), Faculty of Health Sciences, University of the Witwatersrand, Johannesburg, South Africa; 6 Health Systems Research Unit, South African Medical Research Council, Bellville, South Africa; 7 African Social Sciences Unit of Research and Evaluation (ASSURE), Wits Health Consortium, Johannesburg, South Africa; 8 Centre for Health Policy, School of Public Health, University of the Witwatersrand, Johannesburg, South Africa

**Keywords:** COVID-19, Adolescents, Youth, South Africa, Health equity

## Abstract

**Background:**

In South Africa, pervasive age and gender inequities have been exacerbated by the COVID-19 pandemic and public health response. We aimed to explore experiences of the COVID-19 pandemic among youth in eThekwini district, South Africa.

**Methods:**

Between December 2021-May 2022 we explored experiences of the COVID-19 pandemic on youth aged 16–24 residing in eThekwini, South Africa. We collated responses to the open-ended question *“Has the COVID-19 pandemic affected you in any other way you want to tell us about?”* in an online survey focused on understanding the pandemic’s multi-levelled health and social effects. We used a thematic analysis to summarise the responses.

**Results:**

Of 2,068 respondents, 256 (12.4%, median age = 22, 60.9% women) completed the open-ended survey question (11% in isiZulu). Results were organized into three main themes encompassing (1) COVID-19-related loss, fear, grief, and exacerbated mental and physical health concerns; (2) COVID-19-related intensified hardships, which contributed to financial, employment, food, education, and relationship insecurities for individuals and households; and (3) positive effects of the pandemic response, including the benefits of government policies and silver linings to government restrictions.

**Conclusions:**

We found that South African youth experienced significant grief and multiple losses (e.g., death, income, job, and educational) during the COVID-19 pandemic. Trauma-aware interventions that provide economic and educational opportunities must be included in post-COVID recovery efforts.

**Supplementary Information:**

The online version contains supplementary material available at 10.1186/s12889-024-19238-7.

## Introduction

The COVID-19 pandemic has devastated people’s health, education, and economic security and has exacerbated existing inequalities and vulnerabilities [1–3]. Adolescents are at a critical stage of development and may face long-term consequences due to disruptions in education and social support [4–6].

Pandemic responses initially overlooked the effect on youth, not considering them high-risk compared to older or medically vulnerable groups [7]. However, the indirect, multidimensional effects of the pandemic on youth are profound, including immediate and long-term socioeconomic impacts [3, 7]. These effects include increased child poverty, worsened learning conditions, threats to child health from economic hardship, safety concerns, and worsening mental well-being [2, 8]. The pandemic’s disruption to youth’s access to education and employment opportunities is likely to place them on a volatile trajectory in finding and maintaining quality jobs and income [9]. Pre-pandemic, young people faced challenges to a successful future with a limited labour market, lack of affordable housing, and difficulty accessing their rights [7, 10]. The far-reaching effects on youth may have enduring consequences for society [8], with youth and future generations bearing significant long-term economic and social burdens worldwide [1, 9, 11].

In South Africa, where the majority of the population (62.6%) was living below the poverty line pre-pandemic [12], research has shown that food insecurity and hunger worsened among young people [13]. This has particularly severe implications for youth, who may face multiple losses and challenges during this critical period of physical, cognitive, and social development [5, 6]. There have been reports of mental health concerns among South African youth [13]. Moreover, the unemployment rate among young people aged 15–34 was 46.3% in 2021, with youth disproportionately accounting for 59.5% of total unemployed persons [14]. Prolonged periods of unemployment in youth have long-lasting effects on income and mental health, occurring beyond the period of economic recession, as well as risks of concurrent and future insecure employment [7, 9].

Despite the broad scope of literature on the effects of the COVID-19 pandemic, there is a gap in knowledge on the experiences and impact of the pandemic on youth in sub-Saharan Africa. To address this gap, our team launched the AYAZAZI RIGHTS (**R**apid **I**nvestigation of **G**endered **H**ealth outcomes in the **T**ime of **S**ARS-CoV-2) survey in 2021 to explore the multileveled effects of the COVID-19 pandemic on youth overall and by gender. As part of the survey, we included an open-ended question to gather qualitative data on the experiences of youth in eThekwini, South Africa during the COVID-19 pandemic.

By providing an open-ended comment box, we sought to create a space where youth could openly express their thoughts, feelings, and concerns in their own words without being constrained by pre-determined response options [15, 16]. By recognising and valuing youth living experience, we aim to provide a more comprehensive and accurate understanding of the pandemic on youth. This may inform the development of effective and equitable interventions and policies. By shedding light on youth’s unique needs and vulnerabilities, this study seeks to inform interventions to support the resilience and recovery of young people in South Africa.

## Methods

### Study setting

As of August 2nd, 2023, South Africa had the highest recorded number of COVID-19 cases in the African region, accounting for 43% of all cases [17]. KwaZulu-Natal is the second most populous province in South Africa, with the second highest number of confirmed COVID-19 cases at 728,708 in February 2023 [18–20]. eThekwini, being the most populous district in KwaZulu-Natal, is home to some 3.5 million people [21], with youth aged 15–24 years-old comprising 16.1% of the population (731,267) [22]. eThekwini district had recorded the highest number of cases in the province, accounting for 49% of all cases and 35% of deaths in the province in May 2022 [19] and recording 358,222 cases and 5,707 deaths by February 2023 [20].

South Africa underwent a national lockdown beginning March 27th, 2020, considered the most restrictive lockdown in Sub-Saharan Africa [23]. Restrictions included a stay-at-home order, limited gatherings, alcohol and tobacco sale bans, and travel restrictions [24]. Varying lockdown restrictions followed this in response to multiple waves of COVID-19 infection levels from May 2020 to April 2022, when the National State of Disaster was terminated.

From December 21st, 2021 to May 31st, 2022, data from online surveys were collected to explore the multi-levelled effects of the COVID-19 pandemic on youth aged 16–24 in the eThekwini district of KwaZulu-Natal, South Africa.

### Study design

Youth aged 16–24 years, living in the eThekwini district, Durban, South Africa, who could read in English and/or isiZulu and had access to a mobile phone, tablet, or computer that could access the internet were eligible for participation. The survey included questions on socio-demographics, COVID-19 experiences (e.g., illness, deaths, vaccination) and impact (e.g., job, income, food access), as well as other sexual, reproductive, mental health, and substance use experiences and effects from the start of the COVID-19 pandemic to the time of the interview. The final survey question allowed participants to choose to enter free text to the question: “Has the COVID-19 pandemic affected you in any other way you want to tell us about?”

The overall AYAZAZI RIGHTS study was a mixed method design, capturing quantitative and qualitative data through an online survey. This analysis used qualitative methods to analyse responses to an open-ended text comment box. Our analysis was informed by a youth-centered and participatory approach, which sought to prioritise youth voices and perspectives and value their lived experiences. Online qualitative surveys hold the advantages of being able to capture diverse perspectives, experiences, and sense-making from a wide population [25]. The feeling of anonymity encourages disclosure and participation for sensitive topics [26].

Analysing open-ended data offered valuable insights [16]. Unlike closed survey responses, open-ended questions elicit more comprehensive information, allowing participants to provide perspectives beyond researchers’ potential biases [16]. Open-ended data can uncover overlooked issues, enhancing the study’s overall understanding. Additionally, sharing participants’ own words on the topic is beneficial for informing decision-makers and shaping effective, equitable interventions and policies [16]. In the case of our study, the thematic analysis of the open-ended responses provided a deeper understanding of the effects of the COVID-19 pandemic on youth in eThekwini district, South Africa, than the quantitative survey responses alone. As such, our analysis was able to delve further into reasons why young people’s mental health was so affected by the pandemic.

### Data collection- recruitment

We recruited participants for our study through diverse methods, including community organisations, advisory boards, social media, and flyers placed in youth frequented areas like retail spaces and transit hubs. We also engaged previous eligible participants from other studies at the Maternal Adolescent and Child Health Research Unit (MRU). To enhance participation, those who completed the full mobile survey could enter a cash prize draw of R100 (CAD$ 8.50), with winning odds varying from one in five to one in twenty during the enrollment period. Participants accessed the survey via a cost-free web link provided through the #datafree Moya Messenger App.

### Data analysis

This study qualitatively analyzed open-ended responses on the effects of COVID-19 in English or isiZulu. Responses like “not applicable” or with limited detail, such as a simple “no,” were excluded. We translated isiZulu comments to English and merged them into a single dataset for analysis in NVivo 12. In NVivo, we used thematic analysis to explore the experiences of youth in eThekwini district, South Africa, during the first 26 months of the COVID-19 pandemic. This approach, chosen due to global limited and rapidly evolving pandemic evidence, provided flexibility to highlight youth voices and their experiences while placing them within the broader literature and context [27].

Both KC and ED initially reviewed all comments to familiarize themselves with the data. Insights from this process were then shared with the team. KC initiated the coding process by reviewing each comment and grouping into initial ‘codes’. Together KC and ED defined initial codes and ED reviewed for accuracy and alignment with code definitions. Initial codes were shared with the remaining team and through iterative discussions, codes evolved into broader categories, which were eventually distilled down to three overarching themes. This iterative theme refinement process allowed team members to gain insight from the data and provided opportunity for multiple members of the team’s perspective to be considered throughout the analysis [28]. After multiple group discussions. We selected exemplary quotes to highlight the identified themes and ensure representation across age and gender. Only a small number of non-binary participants responded to the open-ended question (< 5). Thus, to protect their identity, we have excluded non-binary participants socio-demographic information from the results below. The term non-binary refers to people whose gender identities and expressions do not conform to binary understanding of gender [29].

### Ethics

Ethical approval was provided by the Simon Fraser University Research Ethics Board and the University of British Columbia Behavioural Research Ethics Board (REB number: H21-02027), and by the University of the Witwatersrand Human Research Ethics Committee (Wits HREC-Medical) in South Africa (REB number: M210863). Participants were provided with an electronic consent letter, detailing the purpose of the survey, benefits and risks for participation, and key contacts for further question before accessing the questionnaire. A list of resources including online and in-person local support services such as mental health and sexual and reproductive health care was provided at the end of the questionnaire. All participants names are pseudonyms to protect confidentiality.

## Results

Of 2,068 participants with non-duplicate responses and complete gender data, 353 (17.1%) included comments in a survey comment box. 97 of these comments were removed as they reported “no” or “NA” without further explanation. Thus, 256 youth (12.4% of the enrolled sample, median age = 22, 60.9% women) submitted a comment (11% in isiZulu). Differences between those who did and did not answer the comment box can be found in Supplementary Material. Additional gender-stratified demographics of youth who submitted a can be found in Table 1. Our results were organised into three themes based on an iterative theme refinement process, emergent from the coding process. Together, these three themes help reflect the diversity of experiences affecting eThekwini youth during the pandemic. They include: (1) Loss, fear, grief, and exacerbated mental and physical health concerns; (2) Heightened financial, educational, or other insecurities; and (3) Positive effects and silver linings of the pandemic response.

**Table 1:** Demographics of South African young women and men who filled out the open-ended comment box (*n* = 253)

	Women (*n* = 156)	Men (*n* = 97)
Age (Median Q1, Q3)	22 (20, 23)	22 (19, 23)
Age group		
16–18	19 (12.2)	14 (14.4)
19–21	55 (35.3)	31 (32.0)
22–24	82 (52.5)	52 (53.6)
LGBTQ+: Yes	30 (19.2)	16 (16.5)
Language: Zulu	17 (10.9)	11 (11.3)
Race		
Black	125 (80.1)	80 (82.5)
Coloured	28 (18.0)	11 (11.3)
Indian	< 5	< 5
White	< 5	< 5
Any Children: Yes	85 (54.8)	37 (38.1)
In Relationship: Yes	127 (81.9)	75 (77.3)
COVID diagnosis: Yes	56 (35.9)	39 (40.2)
COVID Vaccinated: Yes	110 (70.5)	59 (60.8)
In school or employed: Yes	114 (73.1)	64 (66.0)

LGBTQ+, any participants identifying as lesbian, gay, bisexual, non-binary, trans, questioning, or did not identify with any gender and/or sexual orientation

### Theme 1: COVID-19-related loss, fear, grief, and exacerbated mental and physical health concerns

Theme one was most common theme discussed in the comments and consists of two sub-themes: ‘loss’ and ‘fear, grief, and exacerbated mental health concerns’.

#### Loss

Much of our data illustrated how common an experience it was for a youth in eThekwini district to have known someone, whether a relative or community member, who died of COVID-19. Loss was highlighted through general comments that ‘COVID-19 was a death sentence’ (Themba, age 21, woman) to much more detailed descriptions of grief and loss that were experienced equally across age and gender. Many young people spoke not just about acquaintances or neighbours, but of members of their immediate families dying from COVID-19. For example:

It [COVID-19] affected me deeply because I lost my father to COVID. -Nia; age 19; woman

#### Fear, grief, and exacerbated mental health concerns

While many of the comments simply described the experience of losing someone to COVID-19, others were more detailed in describing the grief experienced from losing a loved one to COVID-19. For example, Nkosi highlighted the fear and grief he experienced in losing a relative while many others were dying:

[I] Felt very scared during the first [wave of] COVID-19. […] many people dying from it [COVID-19], hospitals were fully packed, and doctors and nurses were overworked. Many people dying. Lost a relative to COVID, it was very sad and traumatising. - Nkosi; age 20; man

The trauma of losing a loved one was also amplified by the loss that everyone in youths’ communities were also experiencing. Beyond these experiences of having a close family member die of COVID-19, youth also spoke of the trauma of getting very sick from the virus. Getting COVID-19 in the context of high mortality was scary for many participants, as they feared that they might pass COVID-19 to a family member. One participant stated:

I had to stay at home because I was very ill. It was very traumatic for me being sick and many people dying of COVID. - Sbusiso; age 22; man

The onset of COVID-19 illness added an additional layer of complexity to the capacity of numerous young people to work, provide for their families, and heightened concerns about spreading the infection to others.

Finally, mental health concerns were heightened due to public health restrictions that further prevented youth from working, as exemplified by Amahle:

During the COVID-19 pandemic I lost my job due to the pandemic. […] This led me to depression because I couldn’t help my family with groceries and the thing we need. Worse part I had to explain myself to my 7 years old son why I hardly have money for […] his lunch box […]. I got depressed and started to do drugs (weed) […]. Lucky I still have my mom who helped me with most of the things.” - Amahle; age 23, woman

The above quote highlights how the financial strain imposed by pandemic regulations and restrictions was a source of emotional distress, leading to depression. This distress was compounded by the necessity of conveying their financial difficulties to their child. Additionally, the quote mentions the use of substances as a coping mechanism to address the distress and underscores the significance of the family as a support system. In its entirety, the quote serves as a poignant narrative that highlights the interwoven themes of fear, grief and exacerbated mental health concerns within the context of an unprecedented global health crisis.

### Theme 2: COVID-19 intensified hardships contributed to financial, employment, food, educational, and relationship insecurities

This theme describes the enhanced socioeconomic hardships experienced during the pandemic and consists of three sub-themes: ‘harder life’, ‘worry about the future’, and ‘the effect of hardships on relationships’.

#### Harder life

Youth shared the overlapping hardships that they experienced during the pandemic. Many comments left by participants were brief, stating how life was hard or how the pandemic changed everything, such as broad comments like ‘*life became harder with COVID-19’* (Ndlovu; age 20; man). Some individuals provided more detailed insights, delving into how the pandemic worsened economic uncertainties at individual and household levels. For example, comments spoke of specific hardships including youth losing their jobs, their family members losing work, or the household struggling to meet ends meet. Young men were more likely to discuss their own individual struggles with losing work or challenges in securing employment. For example:

I couldn’t find a job because most companies were not performing well. I tried to invent a small business, but I did not have enough capital to implement my ideas. – Tebello; age 23; man

While young women also discussed their own challenges with employment, they were more likely to mention additional hardships facing household members. For example, Amara stated:

Yes, it (COVID-19) has (affected me) because now I’m not working and even my sister too, so no one is working at home now. -Amara; age 24; woman.

Although access to employment was limited and many individuals were facing a reduced income, participants also highlighted that food was hard to come by and the food that was available was more expensive than before the pandemic. For example, Makena stated:

Yes, it (COVID-19) affected us, we could not go to the shops. We were limited things we buy in store and couldn’t go to school every day. Our parents were retrenched. Things were not the same in the house as food went up. – Makena; age 22; woman

#### Worry about the future

The comments also echoed concerns regarding the future, highlighting how the pandemic intensified pre-existing gaps in career opportunities and disrupted crucial stages of young people’s education. For example, Ndlovu stated:

Going to campus stopped and everything was online. I couldn’t find any part time work while I was studying, and I can’t find work now no matter how much I try. I can’t find work outside the country because I can’t afford to travel. I am seriously worried about the damage the pandemic has done to the economy and my future as well as the future of my friends who share the same challenges. – Ndlovu; age 23; man

Youth, especially adolescent girls and non-binary participants highlighted the insecurities to education that the pandemic brought. Accessing online education without adequate internet connections or proper technology led many young people to miss many educational experiences. For example:

School attendance has been a problem since we’re not attending every day and my grades have dropped. -Omphile; non-binary

#### Hardships effects on relationships

Both young men and women talked about how the loss of job or income because of the pandemic affected their relationships. For example, Zola stated:

My partner lost his job and couldn’t support me anymore, that made us fight a lot. - Zola; age 18; man

Another participant ‘Ada’ similarly stated:

My partner lost his job, and I am selling food and fruits in the streets, so my partner when he is angry, he hit me, but now [it is] better. - Ada; age 22; woman

Economic insecurities and lack of employment opportunities for young men was commonly discussed by young men and their partners.

### Positive effects of the COVID-19 pandemic

Finally, while government restrictions were mostly discussed negatively, some youth, particularly young women, spoke of the positive effects that they experienced, considering the challenges raised by the COVID-19 pandemic. These positive effects included the benefits of government policies and silver linings to government restrictions. For example, one participant, Lindiwe, discussed how increases in social grant support during COVID were helpful:

It was a bit hard at home, but at least the grant money was raised so it also helped. -Lindiwe, age 20, woman

Similarly, another young woman, Zuri, highlighted how beneficial the COVID relief grant was for them:

Yes. I would say it [the pandemic] has affected me positively as I am unemployed but managed to have an income of R350 from the government. This R350 is making a huge impact as I am able to buy cosmetics and assist where I can at home as there is no one who is working currently. –Zuri, age 22, woman

Another participant, Busi, discussed the positive effect that online learning had on them:

In a good way sometimes in terms of school studying online has helped because I watch the video multiple times for me to understand rather than being in a lecture hall it’s hard for me having to listen/study there, COVID has [taught us] to appreciate life and has taught the importance of listening to instructions to keep yourself and those around you safe. -Busi, age 21, woman

These quotes highlight the resilience of youth in the face of adversity. They may also highlight other ways that the pandemic disproportionately affected or privileged different groups of youth based on income and other sources of support.

## Discussion

The three themes identified in our study reflect the diverse and complex ways the COVID-19 pandemic has affected the lives of young people in eThekwini. The experiences mentioned by participants in our study are congruent with many of the experiences of the COVID-19 pandemic highlighted in the available literature on South African youth [13, 30–32] and low-and-middle-income (LMICs) youth experiences [7, 33, 34] where the effects on mental health, education, and socio-economics have been key findings [35].

Our results provide insights into the mental health effects of the COVID-19 pandemic on South African youth, aligning with existing research on the subject [36, 37]. Furthermore, results highlight how these mental health declines are likely influenced by the amount of loss experienced during the COVID-19 pandemic. These negative mental health outcomes include not only the grief and trauma associated with COVID-19 related deaths, but also the secondary effects on economic, job, and educational insecurities due to the restrictions imposed by the pandemic. Disenfranchised grief, relating to unacknowledged loss [38, 39], adds a unique dimension, particularly in LMICs like South Africa, where traditional mourning practices that hold significant cultural and social significance were disrupted due to pandemic restrictions [38, 39].

Many youth in our study lost caregivers, potentially disrupting their support systems and exacerbating mental health challenges [40]. Additionally, the pandemic heightened socio-economic instability, most notably food insecurity, particularly affecting urban youth [32]. Addressing these issues, including limited job opportunities and food insecurities, is crucial for mitigating the pandemics’ negative effects on youth mental health.

Our findings underscore the significant emotional toll that the pandemic has taken on young people, with many reporting increased levels of fear, stress, anxiety, and depression. The mental health issues raised by youth in our study align with concerns raised by South African healthcare workers and underscore the need for improved mental health support for youth. Mental health vulnerabilities among youth in our study are especially concerning as mental health issues and linked sexual and reproductive health concerns among youth in South Africa were already high pre-pandemic [41–43].

Despite the hardships, some youth reported positive experiences, such as thriving in online learning environments and a newfound appreciation for life during lockdown. These instances highlight the resilience of South African youth and point to areas of post-traumatic growth that have been seen in the context of the pandemic among young people across the globe [44, 45]. However, results should be interpreted with caution as it is likely that the positive effects to online learning were only available to more well-off youth who had access to stable and consistent internet.

### Implications and recommendations

These findings underscore the profound and extensive effects of the COVID-19 pandemic on South African youth, offering valuable insights to guide efforts in supporting and safeguarding this vulnerable demographic. Moreover, these insights have significant implications for public health, particularly in understanding the pandemic’s effect on youth health and wellbeing in eThekwini. The pandemic worsened pre-existing mental health conditions and gave rise to more, underscoring the importance of ensuring that young people can access the necessary support and resources. This could include therapy, counselling, support groups, education and awareness campaigns that promote mental health and wellness [46, 47]. Additionally, recognising that trauma and grief significantly contribute to mental health issues among youth, tailored trauma-aware^1^ approaches are crucial [48]. Furthermore, as a substantial portion of these mental health repercussions stem from heightened socioeconomic instability, public health policies and interventions should prioritise economic revitalisation for youth in the region. These policies and interventions include financial support for youth and their families, education and career development programs, and initiatives promoting health and wellness, including access to nutritious food [49], physical activity [50], and comprehensive support [51, 52]. Twenty-eight million South Africans, nearly half of the population (47%), receive social grants, with 10 million South Africans age > 18 years old receiving the COVID-19 special relief grant of R350 monthly [53]. Available financial support to South African youth is crucial to South Africa’s development with young people aged 18–34 constituting almost a third of the population [54]; being the next working generation, their health and wellbeing is vital.

### Strengths and limitations

Several strengths and limitations of our study should be noted. A strength is that even though the open-ended study question was presented at the end of a relatively lengthy survey, many youth still wrote detailed responses. This analysis of open-ended text responses allowed our team to capture detailed and nuanced experiences of youth, highlighting the importance of analysing such data.

There are several limitations to our study methodology. While analysing and presenting participants’ words shared in questionnaire data is an essential element of community-based research practices, the analysis of the free-form text is limited in that it does not allow for the use of specific measures typically used by qualitative researchers to enhance rigour. For example, we could not explore youth perceptions using live prompts, which is a common practice in collecting qualitative interview data [55]. Use of live prompts help to enhance the fidelity of researchers’ interpretations of the data to meanings originally intended by participants. Additional research would benefit from longitudinal follow-up studies that could help expand on comments and experiences raised by respondents in our study using focus-groups or interviews to substantiate and broaden the insights presented in this paper. Furthermore, efforts are needed to explore the long-term effects of grief experienced by youth during the pandemic. Additional research on ensuring access to health and social protection during pandemics is vital.

## Conclusions

Our findings shed light on the profound grief and hardships experienced by youth in eThekwini due to the COVID-19 pandemic, encompassing both direct losses from family members’ COVID-19 deaths and the subsequent mental health effects resulting from pandemic-induced socioeconomic consequences. These insights contribute to the existing literature on COVID-19’s mental health effects on South African youth. Results emphasise the need for comprehensive, youth-centred approaches to addressing economic and mental health challenges within their unique socioeconomic, cultural, and historical contexts. By considering these findings, public health professionals and policymakers can develop programs and policies that support and protect the health and well-being of this vulnerable population.

### Electronic supplementary material

Below is the link to the electronic supplementary material.

Supplementary Material 1

## Data Availability

The de-identified data cannot be publicly shared as we do not have approval from the community or research ethics board. Researchers or trainees who wish to access the data should contact Dr. Angela Kaida (angela_kaida@sfu.ca) to request access.

## Footnotes

1 Here we use the terminology of “trauma aware” rather than the perhaps more commonly known phrasing of “trauma-informed”. As others have noted, the language of “Trauma aware” reflects humility and understanding that even those who are not experts in addressing trauma can still practice in sensitive ways that demonstrate an awareness of the pervasiveness of trauma experiences among key populations and pursue strategies that centre physical, psychological, emotional safety.

## Abbreviations

LMIC: Low and middle-income country

## References

[1] Lambert H, Gupte J, Fletcher H, Hammond L, Lowe N, Pelling M (2020). COVID-19 as a global challenge: towards an inclusive and sustainable future. Lancet Planet Health.

[2] UNESCO. UNSDG _ policy Brief_ Education during COVID-19 and beyond. UNITED NATIONS; 2020.

[3] United, Nations. Highlights 2021–2022: towards Sustainable Development for all New York. New York department of economic and social Afairs Afairs DoEaS; 2022.

[4] Desmond C, Sherr L, Cluver L. Covid-19: accelerating recovery. 10.1080/1745012820201766731. 2020;16(1):1–6.

[5] Patton GC, Sawyer SM, Santelli JS, Ross DA, Afifi R, Allen NB (2016). Our future: a Lancet commission on adolescent health and wellbeing. Lancet.

[6] Sawyer SM, Afifi RA, Bearinger LH, Blakemore SJ, Dick B, Ezeh AC (2012). Adolescence: a foundation for future health. Lancet (London England).

[7] Rogobete E. COVID-19 Youth Survey: Report. Global Shapers Community, The International Federation of Medical Students’ Assocaitions, Health and Information Literacy Access Alliance. 2020.

[8] United Nations. UN Secretary-General’s policy brief: The impact of COVID-19 on women | Digital library: Publications | UN Women – Headquarters. 2020.

[9] Cazes S, Hijzen A, Saint-Martin A. Measuring and Assessing Job Quality. 2015.

[10] Puerto S, Gardiner D, Bausch J. Youth and COVID-19: impacts on jobs, education, rights and mental well-being: survey report 2020. 2020.

[11] Baird S, Seager J, Tauseef S (2021). The effect of COVID-19 on Economic Participation and Human Capital Development of Youth Living in Urban Slums in Bangladesh.

[12] The World Bank. Global Financial Inclusion (Global Findex) Database (Global Findex) | Data Catalog. 2022.

[13] Gittings L, Toska E, Medley S, Cluver L, Logie CH, Ralayo N et al. ‘Now my life is stuck!’: Experiences of adolescents and young people during COVID-19 lockdown in South Africa. https://doiorg/101080/1744169220211899262. 2021;16(6):947 – 63. 10.1080/17441692.2021.1899262 PMC10105067 33750269

[14] Statistics South Africa (2021). General Household Survey 2019.

[15] O’Cathain A, Thomas KJ (2004). Any other comments? Open questions on questionnaires - a bane or a bonus to research?. BMC Med Res Methodol.

[16] Decorte T, Malm A, Sznitman SR, Hakkarainen P, Barratt MJ, Potter GR et al. The challenges and benefits of analyzing feedback comments in surveys: lessons from a cross-national online survey of small-scale cannabis growers. Methodological Innovations. 2019;12(1).

[17] World Health Organization. COVID19 Cases 2023 https://who.maps.arcgis.com/apps/dashboards/0c9b3a8b68d0437a8cf28581e9c063a9.

[18] National Institute for Communicable Diseases. Weekly Epidemiological Brief - NICD 2022 https://www.nicd.ac.za/diseases-a-z-index/disease-index-covid-19/surveillance-reports/weekly-epidemiological-brief/.

[19] Lee J-Y, Tsai C-S, Hsu C-C, Khambule I. Territorial Impact and Responses to COVID-19 in South Africa: Case Studies of eThekwini Metropolitan Municipality and KwaDukuza Local Municipality. World 2022, Vol 3, Pages 513–529. 2022;3(3):513 – 29.

[20] KZN Health. Coronavirus (COVID-19) 2023. https://www.kznhealth.gov.za/coronavirus.htm.

[21] Statistics South Africa (2019). Mid-year Population estimates in: statistics, editor.

[22] Africa SS, Statistics South Africa (2022). Mid-year Population estimates.

[23] Stiegler N, Bouchard J-P, editors. South Africa: challenges and successes of the COVID-19 lockdown. Annales Médico-psychologiques, revue psychiatrique. Elsevier; 2020. 10.1016/j.amp.2020.05.006 PMC7250766 32836300

[24] South African Government. COVID-19/Coronovirus 2023. https://www.gov.za/Coronavirus.

[25] Braun V, Clarke V, Gray D, Gough B (2017). Innovations in qualitative methods. The Palgrave handbook of critical social psychology.

[26] Braun V, Clarke V, Boulton E, Davey L, McEvoy C (2021). The online survey as a qualitative research tool. Int J Soc Res Methodol.

[27] Achoki T, Sartorius B, Watkins D, Glenn SD, Kengne AP, Oni T (2022). Health trends, inequalities and opportunities in South Africa’s provinces, 1990–2019: findings from the global burden of Disease 2019 study. J Epidemiol Community Health.

[28] Braun V, Clarke V (2021). One size fits all? What counts as quality practice in (reflexive) thematic analysis?. Qualitative Res Psychol.

[29] Women’s Health Research Institute (WHRI). Beyond the Binary in British Columbia: A Guide. 2024.

[30] Duby Z, Bunce B, Fowler C, Bergh K, Jonas K, Dietrich JJ et al. Intersections between COVID-19 and Socio-economic mental health stressors in the lives of South African adolescent girls and Young women. Child Adolesc Psychiatry Mental Health. 2022;16(1). 10.1186/s13034-022-00457-y PMC8959551 35346316

[31] Rwafa-Ponela T, Price J, Nyatela A, Nqakala S, Mosam A, Erzse A et al. We were afraid: Mental Health effects of the COVID-19 pandemic in two South African districts. Int J Environ Res Public Health. 2022;19(15). 10.3390/ijerph19159217 PMC9368439 35954573

[32] VanVolkenburg H, Vandeplas I, Toure K, Sanfo S, Balde FL, Vasseur L. Do COVID-19 and food insecurity influence existing inequalities between women and men in Africa? Int J Environ Res Public Health. 2022;19(4). 10.3390/ijerph19042065 PMC8871765 35206252

[33] Ogueji IA, Agberotimi SF, Adesanya BJ, Gidado TN (2021). Mental health and coping strategies during the COVID-19 pandemic: a qualitative study of unemployed and employed people in Nigeria. Analyses Social Issues Public Policy.

[34] Ramaiya A, Chandra-Mouli V, Both R, Gottert A, Guglielmi S, Beckwith S (2023). Assessing the health, social, educational and economic impact of the COVID-19 pandemic on adolescents in low- and middle-income countries: a rapid review of the literature. Sex Reprod Health Matters.

[35] United Nations. The Sustainable Development Goals Report 2022. New York; 2022.

[36] Banati P, Jones N, Youssef S. Intersecting vulnerabilities: the impacts of COVID-19 on the psycho-emotional lives of Young people in low- and Middle-Income Countries. Eur J Dev Res. 2020:1–26. 10.1057/s41287-020-00325-5 PMC7649704 33191985

[37] Idele P, Banati P (2020). We are all in this together: COVID-19 and a call to Action for Mental Health of Children and adolescents. Front Psychiatry.

[38] Albuquerque S, Teixeira AM, Rocha JC (2021). COVID-19 and disenfranchised grief. Front Psychiatry.

[39] Kumar N, Janmohamed K, Nyhan K, Forastiere L, Zhang WH, Kagesten A (2021). Sexual health (excluding reproductive health, intimate partner violence and gender-based violence) and COVID-19: a scoping review. Sex Transm Infect.

[40] Roberts KJS, Du Toit S, Mawoyo T, Tomlinson M, Cluver LD, Skeen S (2023). Protocol for the OCAY study: a cohort study of orphanhood and caregiver loss in the COVID-19 era to explore the impact on children and adolescents. BMJ open.

[41] Barhafumwa B, Dietrich J, Closson K, Samji H, Cescon A, Nkala B (2016). High prevalence of depression symptomology among adolescents in Soweto, South Africa associated with being female and cofactors relating to HIV transmission. Vulnerable Child Youth Stud.

[42] Jesson J, Dietrich J, Beksinska M, Closson K, Nduna M, Smit J (2021). Food insecurity and depression: a cross-sectional study of a multi-site urban youth cohort in Durban and Soweto, South Africa. Trop Med Int Health.

[43] Pakhomova TE, Dietrich JJ, Closson K, Smit J, Hornschuh S, Smith P (2021). Intimate Partner Violence, Depression, and anxiety are Associated with higher perceived stress among both Young men and women in Soweto and Durban, South Africa. Front Reproductive Health.

[44] Jian Y, Hu T, Zong Y, Tang W. Relationship between post-traumatic disorder and posttraumatic growth in COVID-19 home-confined adolescents: the moderating role of self-efficacy. Curr Psychol. 2022. 10.1007/s12144-021-02515-8 PMC8736319 35018083

[45] Zhen R, Zhou X (2022). Latent patterns of posttraumatic stress symptoms, Depression, and Posttraumatic Growth among adolescents during the COVID-19 pandemic. J Trauma Stress.

[46] Bradshaw M, Gericke H, Coetzee BJ, Stallard P, Human S, Loades M (2021). Universal school-based mental health programmes in low- and middle-income countries: a systematic review and narrative synthesis. Prev Med.

[47] Ogueji IA, Okoloba MM, Demoko Ceccaldi BM (2022). Coping strategies of individuals in the United Kingdom during the COVID-19 pandemic. Curr Psychol.

[48] Javakhishvili JD, Ardino V, Bragesjö M, Kazlauskas E, Olff M, Schäfer I (2020). Trauma-informed responses in addressing public mental health consequences of the COVID-19 pandemic: position paper of the European Society for Traumatic Stress Studies (ESTSS). Eur J Psychotraumatology.

[49] Dyke E, Pénicaud S, Hatchard J, Dawson AM, Munishi O, Jalal C. Girl-powered Nutrition Program: key themes from a formative evaluation of a Nutrition Program co-designed and implemented by adolescent girls in low- and Middle-Income Countries. Curr Developments Nutr. 2021;5(7). 10.1093/cdn/nzab083 PMC8282357 34286176

[50] Bonnema J, Coetzee D, Lennox A. Effect of a Three-Month HOPSports Brain Breaks^®^ Intervention Program on the Physical Fitness Levels of Grade 6-Learners in South Africa. Int J Environ Res Public Health. 2022;19(18). 10.3390/ijerph191811236 PMC9517318 36141508

[51] Gilberds H, Brown A, Leclair M, Thadzi T, Burnham K. Interactive radio Program Report An Integrated Approach to addressing the issue of Youth Depression in Malawi and Tanzania Acknowledgements. Farm Radio International; 2016.

[52] Thupayagale-Tshweneagae G, Mokomane Z (2014). Evaluation of a peer-based mental health support program for adolescents orphaned by AIDS in South Africa. Japan J Nurs Science: JJNS.

[53] South African Social Security Agency. South African Security Agency Annual Performance Plan 2022–2023. Department of Social Development 2023.

[54] Africa SS. Vulnerability if youth in South African labour market Pretoria, South Africa2020 [updated June 24, 2020. https://www.statssa.gov.za/?p=13379.

[55] Creswell JW, Poth CN. Qualitative inquiry and research design: choosing among five appraoches. 4th ed. SAGE; 2018.

