# Supplementary material for: ‘Life became harder with COVID-19’: exploring the experiences of the COVID-19 pandemic among youth living in eThekwini district, South Africa
Source: BMC Public Health. 2024 Jul 17;24:1922. doi: 10.1186/s12889-024-19238-7 (PMC11256542; doi:10.1186/s12889-024-19238-7)
Supplement: Supplementary file 1 — Supplementary Material 1 [file 12889_2024_19238_MOESM1_ESM.docx]

**Supplementary Material S1- Differences between those who did and did not answer the final comment box in the survey**

|  | **No comments (n=1812)** | **Comments (n=256)** | **P-value*** |
| --- | --- | --- | --- |
| Gender**  Young Man  Young woman | 784 (45.4)  941 (54.6) | 97 (38.3)  156 (61.7) | 0.03 |
| Age group  16-18  19-21  22-24 | 479 (26.4)  411 (22.7)  922 (50.9) | 35 (13.7)  86 (33.6)  135 (52.7) | <0.001 |
| LGBTQ+: Yes | 298 (16.7) | 52 (20.7) | 0.112 |
| Language: Zulu | 55 (3.0) | 28 (10.9) | <0.001 |
| Race  Black  Coloured  Indian  white | 1368 (75.5)  167 (9.2)  190 (10.5)  87 (4.8) | 206 (80.5)  40 (15.6)  <10  <5 | <0.001 |
| Any Children: Yes | 609 (33.7) | 122 (48.0) | <0.001 |
| In a Relationship | 1326 (73.5) | 203 (80.2) | 0.02 |
| COVID diagnosis | 667 (36.8) | 97 (37.9) | 0.643 |
| COVID Vaccinated | 634 (35.0) | 169 (66.0) | 0.198 |
| In school or employed | 1797 (70.6) | 181 (71.5) | 0.749 |

LGBTQ+, any participants identifying as lesbian, gay, bisexual, non-binary, trans, questioning, or did not identify with any gender and/or sexual orientation

*p-values calculated using chi-square test of significance

**Note that non-binary participants were not included in the calculation due to small sample of non-binary participants that completed the open-ended survey question
